# Supplementary material for: Inward- and outward-facing X-ray crystal structures of homodimeric P-glycoprotein CmABCB1
Source: Nat Commun. 2019 Jan 8;10:88. doi: 10.1038/s41467-018-08007-x (PMC6325147; doi:10.1038/s41467-018-08007-x)
Supplement: Supplementary file 1 — Supplementary Information [file 41467_2018_8007_MOESM1_ESM.pdf]

Supplementary Information

Inward- and outward-facing X-ray crystal structures of homodimeric P-glycoprotein CmABCB1

Atsushi Kodan, Tomohiro Yamaguchi, Toru Nakatsu, Keita Matsuoka, Yasuhisa Kimura, Kazumitsu Ueda, Hiroaki Kato

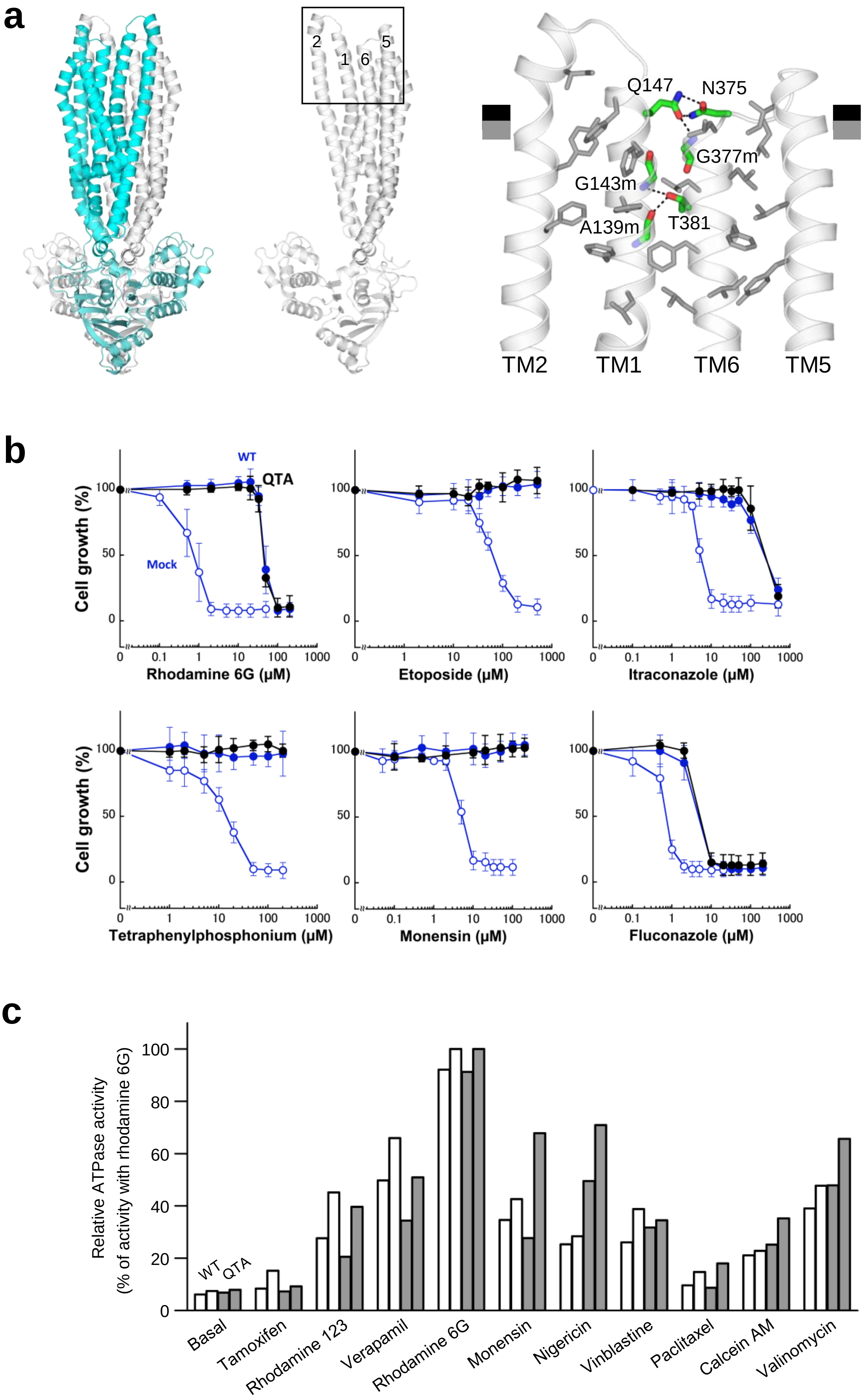

Supplementary Figure 1

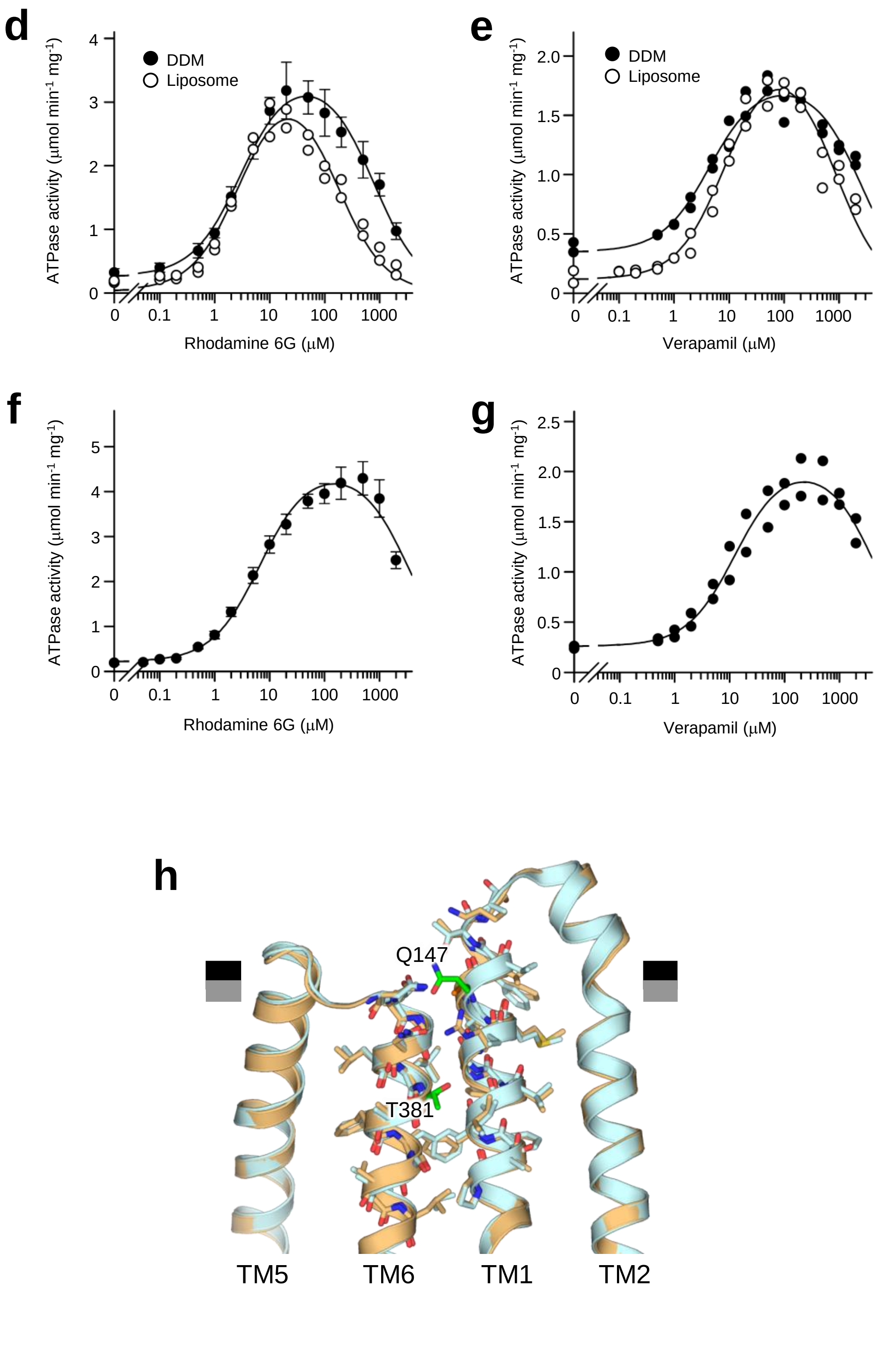

Supplementary Figure 1 (Continued)

Supplementary Figure 1. Structure of wild-type CmABCB1 and functional characteristics of the QTA mutant. (**a**) Wild-type (WT) inward-facing CmABCB1 (PDB code: 3WME) dimer structure (left), monomer structure (middle), and close-up view of the extracellular gate (right). One subunit is shown, and TM3 and TM4 are omitted for simplicity (right). Residues forming hydrogen bonds between TM1 and TM6 are shown as green sticks, and hydrogen bonds are indicated as black dashed lines. Hydrophobic and aromatic residues clustered around the extracellular gate are shown as gray sticks. (**b,c**) Functional characterization of the QTA mutant. (**b**) Drug susceptibility assay in *S. cerevisiae* AD1-8u$^-$ cells expressing QTA CmABCB1. *S. cerevisiae* AD1-8u$^-$ cells expressing QTA CmABCB1 (filled black circles) were grown in the presence of various concentrations of drugs. For each drug assayed, AD1-8u$^-$ cells expressing wild-type CmABCB1 (filled blue circles) or mock-transfected (blue circles) were used as controls. Data are means ± SD (n = 3). (**c**) Drug-dependent ATPase activities of QTA and WT CmABCB1. ATPase activity was measured in the presence or absence of 50 μM of the indicated drugs and 5 mM ATP at 37°C. Two independent data shown as separate bar graphs are expressed as percent of the highest value from the experiment with rhodamine 6G for WT or QTA CmABCB1. (**d–g**) ATPase activity profiles in the presence of various concentrations of rhodamine 6G (d,f) or verapamil (e,g) for WT CmABCB1 (d,e), QTA CmABCB1 (f,g). ATPase activities of CmABCB1 were measured in detergent micelles or liposomes. Two independent data are shown, except data of WT and QTA for rhodamine 6G are means ± SD (n = 3). Data for WT CmABCB1 in detergent micelles (d,e) are from Kodan *et al.*[1]. Drug-dependent ATPase activity of purified CmABCB1 was similar both in detergent micelles and liposomes, whereas that of purified human P-gp depended on reconstitution into liposomes, as shown previously[2,3]. (**h**) Close-up view of superposed WT (cyan) and QTA (orange) CmABCB1. Each subunit is superposed, and the TMDs are shown parallel to the membrane plane. TM3 and TM4 are omitted for clarity. Side chains of residues in TM1 and TM6 are shown as sticks, and the Gln147 and Thr381 residues of WT CmABCB1 are shown in green.

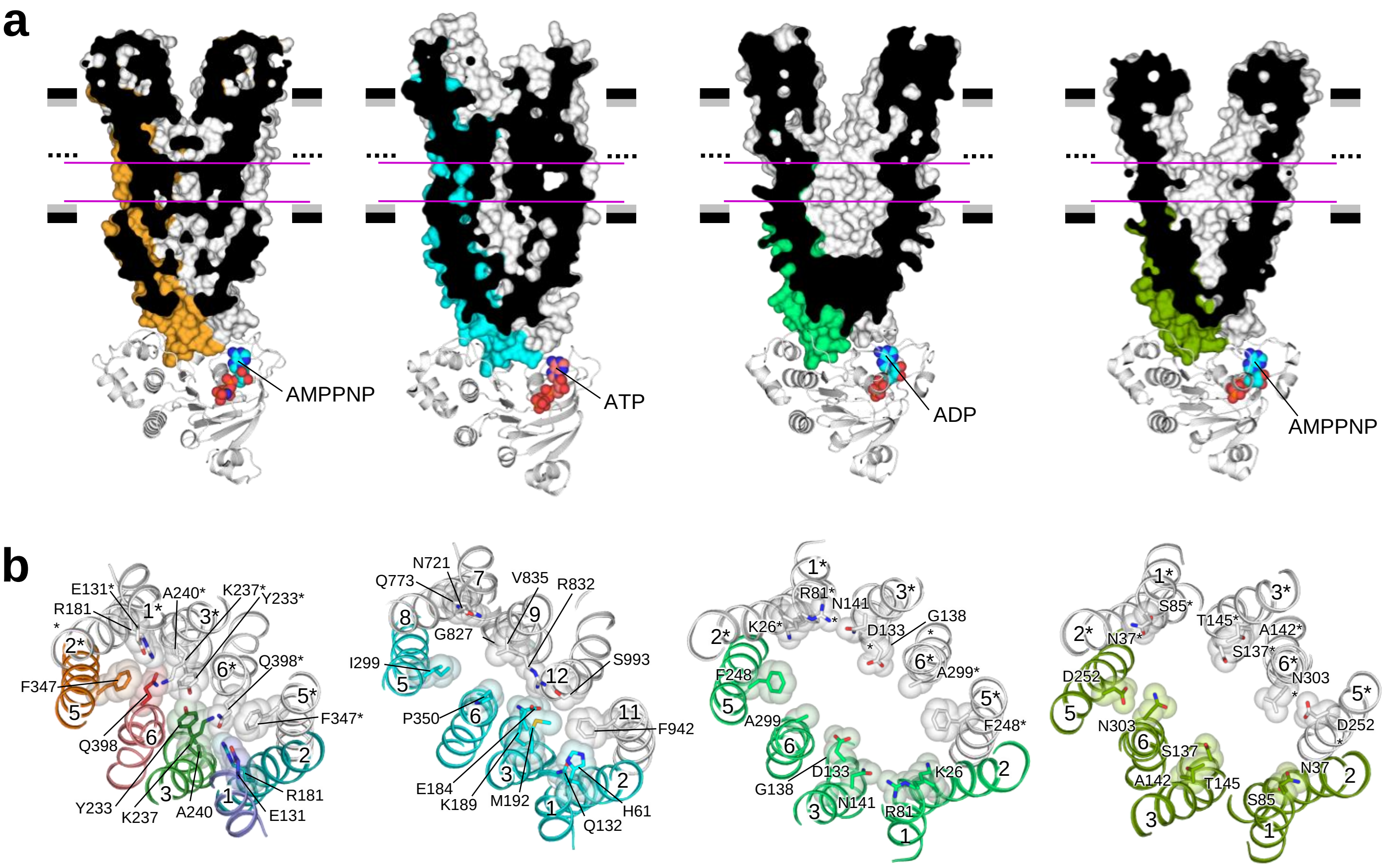

Supplementary Figure 2. Comparison of outward-facing structure of QTA CmABCB1 with human P-gp, Sav1866, and MsbA. (**a**) Cartoon representations of structures of outward-facing QTA CmABCB1, human P-gp (PDB code 6C0V)[4], Sav1866 (PDB code 2HYD)[5], and MsbA (PDB code 3B60)[6], viewed parallel to the membrane as cutaway surface representations of the TMDs, with the interior shown in black. Horizontal black and gray bars represent the expected positions of the hydrophilic and hydrophobic surfaces of the lipid membrane, respectively. (**b**) Cross-sectional view from the extracellular side of outward-facing structures of QTA CmABCB1, human P-gp, Sav1866, and MsbA between the two purple lines shown in (a). Residues of QTA CmABCB1 involved in closure of the space to the cytoplasm and the corresponding residues in human P-gp, Sav1866, and MsbA are shown as spheres, indicating that the chamber is closed in outward-facing QTA CmABCB1 and human P-gp, but wide open in Sav1866 and MsbA.

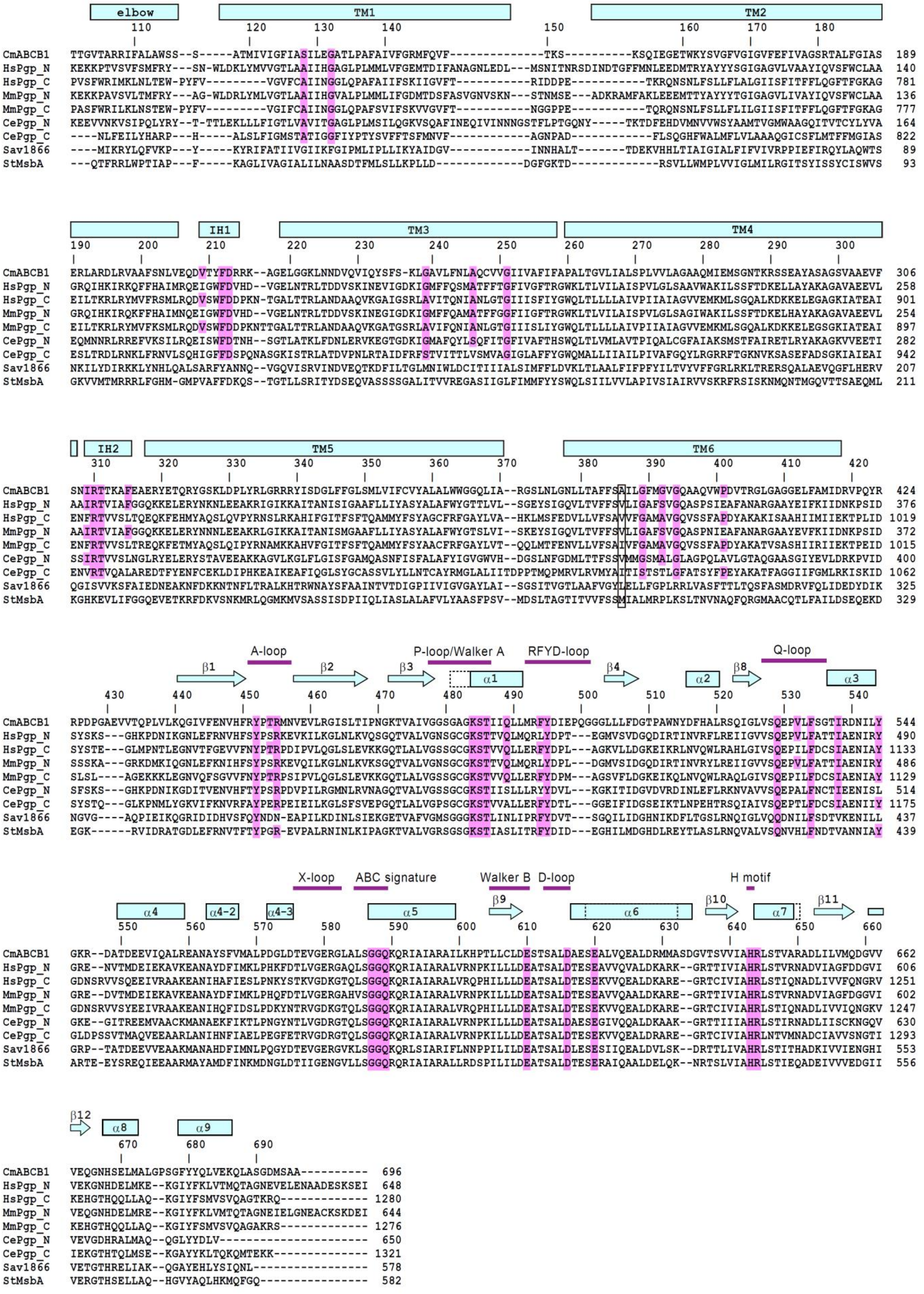

Supplementary Figure 3

Supplementary Figure 3. Amino acid sequence alignment of ABC transporters. CmABCB1 aligned with the N- and C-terminal halves of *H. sapiens* P-gp (GI: 2353263), N- and C-terminal halves of *M. musculus* P-gp (GI: 153791547), N- and C-terminal halves of *C. elegans* P-gp (GI: 17541710), *S. aureus* Sav1866 (GI: 15924856), and *S. typhimurium* MsbA (GI: 16759854). To perform accurate amino acid sequence alignment based on the three-dimensional structures, with manual adjustment, we selected the eukaryotic B-subfamily transporters (*H. sapiens*, *M. musculus*, and *C. elegans* P-gp) for which X-ray or cryo-EM structures have been reported[4,7,8]. For comparison with bacterial homologues, *S. aureus* Sav1866 and *S. typhimurium* MsbA were selected because the X-ray structures of their outward-facing states have been reported[5,6]. The initial alignment was performed using PROMALS3D. Secondary structure elements of CmABCB1 in the outward-facing state are indicated above the sequences. The lengths of α1, α6, and α7 in the inward-facing state are indicated by dotted lines. Residues in TMD conserved among P-gps, including Ala and Ser (which have small side chains and are generally replaced with Gly in the GXXXG motif[9]), as well as NBD residues conserved among B-subfamily transporters described in this report, are highlighted in light magenta. Ala386 (boxed) is not conserved. Known conserved sequence motifs in NBD as well as RE-latch are also indicated. The alignment reveals that the residues participating in the Mg^{2+}-nucleotide–NBD and NBD–TMD interactions are conserved in transporters whose structures have been elucidated.

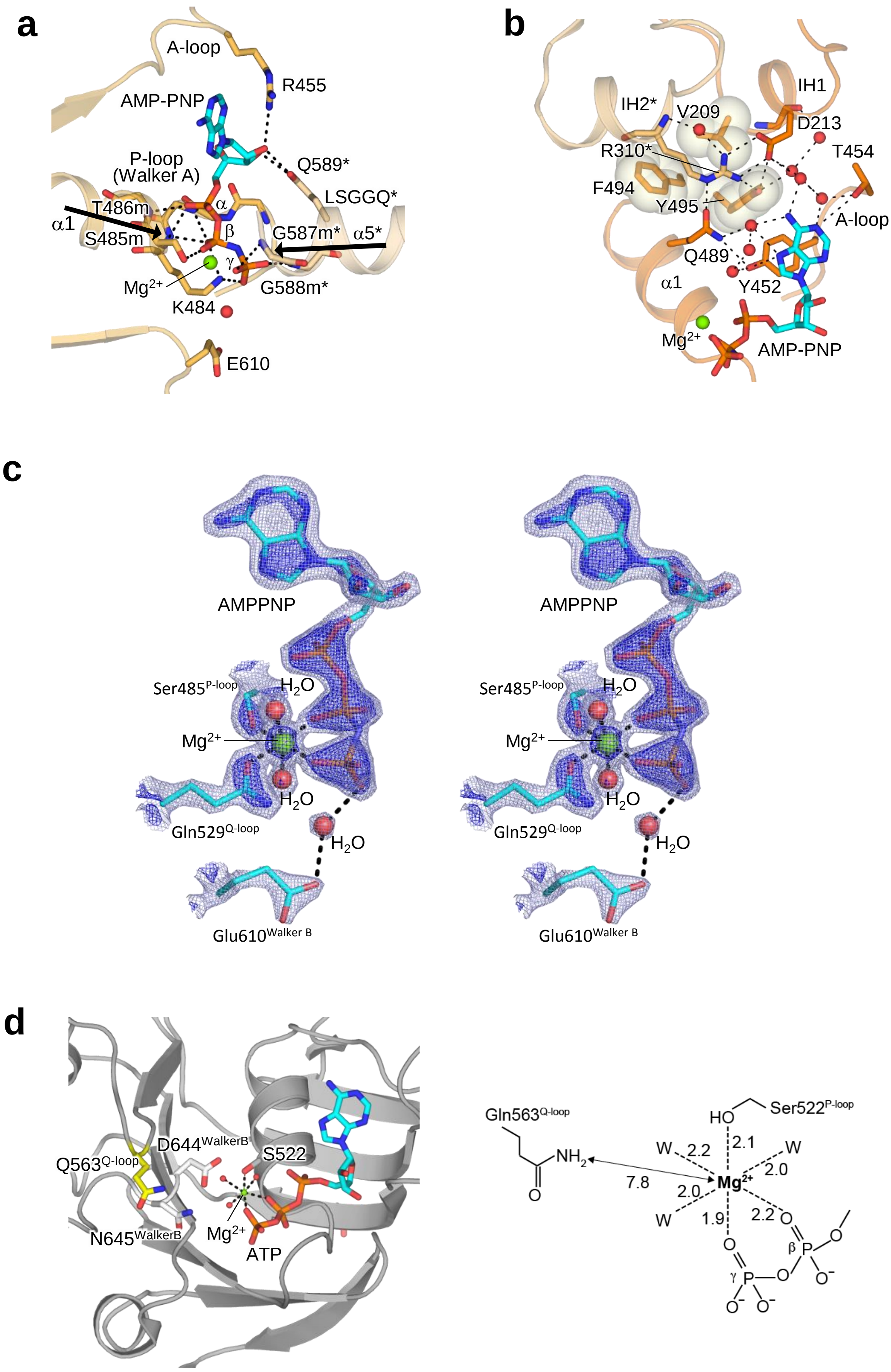
a
A-loop
R455
AMP-PNP
P-loop
(Walker A)
Q589*
LSGGQ*
α
T486m
α1
β
G587m*
α5*
S485m
γ
Mg2+
G588m*
K484
E610
b
IH1
IH2*
V209
D213
R310*
T454
F494
Y495
A-loop
Q489
α1
Y452
Mg2+
AMP-PNP
c
AMPPNP
Ser485P-loop
H2O
Mg2+
H2O
Gln529Q-loop
H2O
Glu610Walker B
AMPPNP
Ser485P-loop
H2O
Mg2+
H2O
Gln529Q-loop
H2O
Glu610Walker B
d
Q563Q-loop
D644WalkerB
S522
N645WalkerB
Mg2+
ATP
Gln563Q-loop
HO
Ser522P-loop
W
2.2
2.1
W
2.0
NH2
7.8
Mg2+
2.0
O
W
1.9
2.2
O
β
P
γ
P
O−

Supplementary Figure 4. Interaction between NBD–NBD' and NBD–TMD. (**a**) AMP-PNP sandwiched by the Walker A and Signature motif. Hydrogen bonds are shown as dashed lines. Mg^{2+} and water molecules are shown as green and red spheres, respectively. Polar interactions are shown as dashed lines. Dipole moments (δ^{+}) of a1 and a5* are shown as arrows. (**b**) Interaction between AMP-PNP and IH helices. Residues $Phe494^{RFYD\text{-}loop}$, $Tyr495^{RFYD\text{-}loop}$, and $Val209^{IH1}$, which form van der Waals interactions, are shown as translucent spheres. Water molecules are shown as red spheres. Polar interactions are shown as dashed lines. The adenine moiety of AMP-PNP is bound with water molecules and fills a space between IH1 and a1 and the A-loop, and interacts strongly with the NBD, IH1, and IH2*. Hydrogen-bonding networks are formed by the adenine moiety of AMP-PNP, $Gln489^{\alpha 1}$, $Thr454^{A\text{-}loop}$, $Tyr452^{A\text{-}loop}$, $Tyr495^{RFYD\text{-}loop}$, $Asp213^{IH1}$, and $Arg310^{*IH2}$ with the aid of water molecules. The H-bonding networks are also stabilized by van der Waals interactions incorporating $Phe494^{RFYD\text{-}loop}$, $Tyr495^{RFYD\text{-}loop}$, and $Val209^{IH1}$. Thus, the H-bonding networks lift IH1, as well as TM3. These structural changes that occur upon ATP binding also assist in chamber contraction, as shown in Fig. 3a,b. (**c**) Stereo view of the octahedral coordination of Mg^{2+}. Light blue and blue meshes represent the 2Fo−Fc map contoured at 2.7 and 4.5 sigma, respectively. Polar interactions are shown as dashed lines. (**d**) Mg^{2+}•ATP binding site in one TAP1-NBD in the homodimer (PDB code 2IXE)[10]. Polar interactions are shown as dashed lines. The distance (7.8 Å) between the Mg^{2+} ion and the nitrogen atom of the $Gln563^{Q\text{-}loop}$ amide group is indicated by a double-headed arrow.

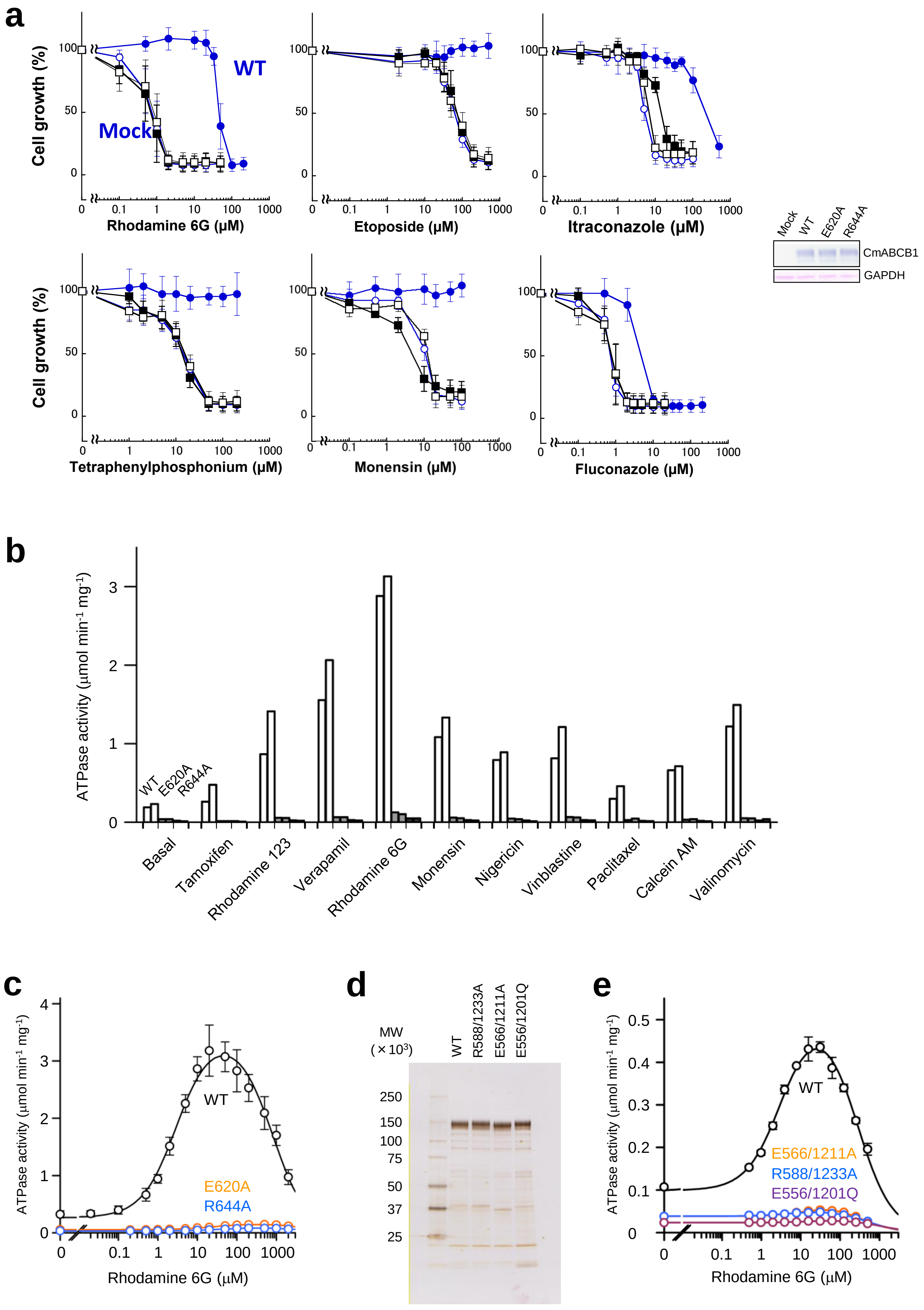

Supplementary Figure 5

Supplementary Figure 5. RE-latch mutants of CmABCB1 and human P-gp. (**a**) Drug susceptibility assay in *S. cerevisiae* AD1-8u$^-$ cells expressing RE-latch CmABCB1 mutants (E620A or R644A). *S. cerevisiae* AD1-8u$^-$ cells expressing E620A (filled black square) or R644A (open black square) were grown in the presence of various concentrations of drugs. For each drug assay, AD1-8u$^-$ cells expressing wild type CmABCB1 (filled blue circle) or mock-transfected (blue circle) were used as controls. Data are means ± SD (*n* = 3). Right panel shows the levels of mutant and WT CmABCB1 protein expressed in AD1-8u$^-$ cells, as determined by Western blotting with anti-His antibody; GAPDH was used as a loading control. Expression levels of the mutants were not affected by their mutations. (**b**) Drug-dependent ATPase activities of RE-latch CmABCB1 mutants (E620A, or R644A) in comparison with wild-type CmABCB1. ATPase activity was measured in the presence or absence of 50 μM of the indicated drugs and 5 mM ATP at 37ºC. Two independent data are shown as separate bar graphs. (**c**) Rhodamine 6G–concentration dependence of ATPase activities of RE-latch mutants and WT CmABCB1. Data are means ± SD (*n* = 3). Data for WT are from Kodan *et al.*[1]. (**d,e**) Human P-gp and its RE-latch mutants (E566/1211A and R588/1233A) expressed and purified from suspension-adapted HEK293 cells (FreeStyle 293F cells) were analyzed by silver staining (d), and their ATPase activities were measured by estimating released ADP (e). A non-catalytic mutant (E556/1201Q), in which the Glu critical for ATP hydrolysis was replaced by Gln was also analyzed. Mutants deficient in RE-latch also had little ATPase activity. Data are means ± SD (*n* = 3).

a

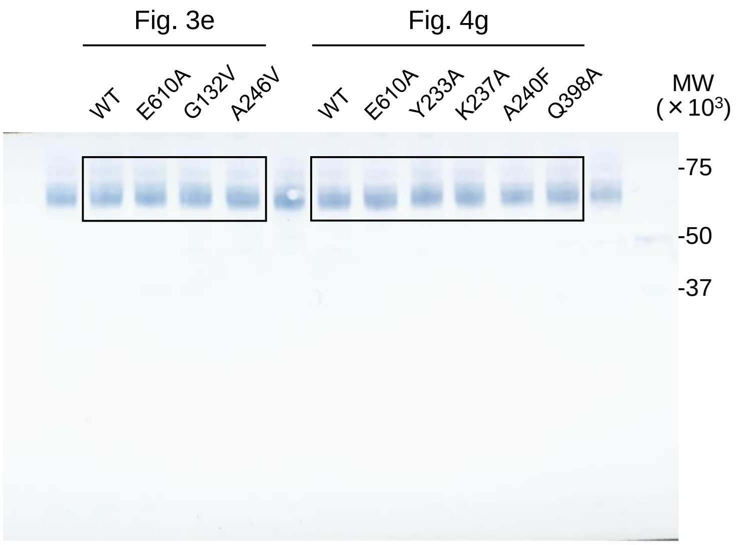

b

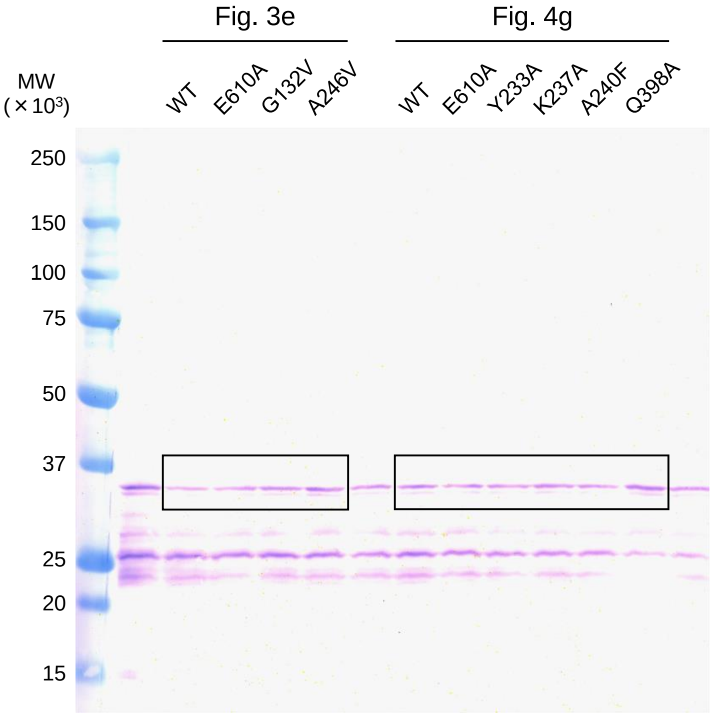

Supplementary Figure 6. Western blotting analysis of the amounts of mutant and WT CmABCB1 in AD1-8u⁻ cells. (**a, b**) Uncropped images of western blots with anti-His (a) or anti-GAPDH (b) presented in Fig. 3e and Fig. 4g are shown.

Supplementary Table 1. Kinetic parameters of ATPase activities of WT and mutant CmABCB1

	Rhodamine 6G					ATP	
	k_{basal} ± s.d. (s^{-1})	k_{sub} ± s.d. (s^{-1})	k_{sub}/k_{basal} ± s.d. (-fold)	K_m^{Drug} ± s.d. (μM)	K_i ± s.d. (μM)	k_{cat} ± s.d. (s^{-1})	K_m^{ATP} ± s.d. (mM)
WT	0.70 ± 0.17	8.9 ± 0.9	13 ± 3	2.9 ± 0.5	800 ± 210	0.84 ± 0.18	0.55 ± 0.02
QTA	0.56 ± 0.06	12 ± 1	21 ± 1	6.7 ± 0.4	3500 ± 410	0.56 ± 0.03	0.28 ± 0.06
Q398A	10 ± 1	2.5 ± 0.3	0.24 ± 0.02	-	1.1 ± 0.3	12 ± 1	0.35 ± 0.03
A240F	14 ± 2	1.6 ± 0.4	0.12 ± 0.02	-	6.5 ± 2.0	19 ± 3	0.30 ± 0.02
G132V	11 ± 3	7.1 ± 2.5	0.64 ± 0.09	-	12 ± 3	16 ± 2	0.091 ± 0.031
A246V	15 ± 2	3.9 ± 0.3	0.28 ± 0.01	-	10 ± 5	19 ± 3	0.29 ± 0.07
E620A	0.14 ± 0.01	0.42 ± 0.03	3.0 ± 0.4	28 ± 6	3800 ± 800	0.14 ± 0.03	0.61 ± 0.05
R644A	0.080 ± 0.029	0.24 ± 0.04	3.3 ± 1.3	39 ± 6	3900 ± 1200	0.073 ± 0.013	0.34 ± 0.15

The values of k_{basal} and k_{cat} for ATP are measured independently. Data are mean ± s.d. from three independent experiments. The data of WT are from Kodan *et al.*[1].

Supplementary Table 2. Strains and primers/oligonucleotides

Name	Description	Source or references
**Strains**		
Saccharomyces cerevisiae		
AD1-8u$^-$ cell	(*Mat a, pdr1-3, his1, ura3, Δyor1::hisG,Δsnq2::hisG, Δpdr5::hisG, Δpdr10::hisG,Δpdr11::hisG, Δycf1::hisG, Δpdr3::hisG,Δpdr15::hisG*)	(11)
Mammalian cell		
FreeStyle 293-F cell		Thermo Fisher
**Primers/Oligonucleotide**		
Construction of CmABCB1 mutants		
Q147A_f	TCGGTCGAATGTTTGCAGTTTTCACGAAGAGC	This study
T381A_f	CTGGGTAATTTGCTAGCAGCATTCTTCAGCGCC	This study
E610A_f	CTCTGCTTGGATGCAAGCACCAGCGCTC	(1)
E610A_r	GAGCGCTGGTGCTTGCATCCAAGCAGAG	(1)
Y233A_f	GTTCAGGTCATCCAGGCTTCGTTCTCGAAAC	This study
Y233A_r	GTTTCGAGAACGAAGCCTGGATGACCTGAAC	This study
K237A_f	CATCCAGTACTCGTTCTCGGCACTCGGTGCGGTTTTATTC	This study
K237A_r	GAATAAAACCGCACCGAGTGCCGAGAACGAGTACTGGATG	This study
A240F_f	CTCGTTCTCGAAACTCGGTTTCGTTTTATTCAATCTCGCC	This study
A240F_r	GGCGAGATTGAATAAAACGAAACCGAGTTTCGAGAACGAG	This study
Q398A_f	GTCGGTCAGGCGGCTGCTGTCTGGCC	This study
Q398A_r	GGCCAGACAGCAGCCGCCTGACCGAC	This study
G132V_f	GCTTCTATTTTGGAAGTTGCAACGCTACCAG	This study
A246V_f	GTTTTATTCAATCTCGTTCAATGCGTTGTCGG	This study
E620A_f	CTGGATGCAGAGAGCGCGGCTCTCGTTCAG	This study
R644A_f	GCGTTGTGATTGCCCACGCTTTGTCCACAGTGGCG	This study
Vector modification for P-gp		
TEV-His-Fw	GGCCGCGGAAAACTTGTACTTCCAAGGTCATCACCATCAC CATCACCATCACCATCACGG	This study
TEV-His-Rv	GGCCCCGTGATGGTGATGGTGATGGTGATGGTGATGACCT TGGAAGTACAAGTTTTCCGC	This study
Construction of human P-gp mutants		
MDR1-R588A-mutF	AGCTCATGCTTTGTCTACAGTTCGTAATGC	This study
MDR1-R588A-mutR	GACAAAGCATGAGCTATCACAATGGTG	This study
MDR1-R1233A-mutF	GCTCACGCCCTGTCCACCATCCAGAATG	This study
MDR1-R1233A-mutR	GGACAGGGCGTGAGCAATCACAATGCAG	This study
MDR1-E566A-mutF	GAAAGCGCAGCAGTGGTTCAGGTGGC	This study
MDR1-E566A-mutR	CACTGCTGCGCTTTCTGTGTCCAAGG	This study
MDR1-E1211A-mutF	GAAAGTGCAAAGGTTGTCCAAGAAGC	This study
MDR1-E1211A-mutR	AACCTTTGCACTTTCTGTATCCAGAG	This study
MDR1-EQEQ-mutF1	TTGGATCAAGCCACGTCAGCTCTGGATAC	This study
MDR1-EQEQ-mutR1	GTGGCCTGATCCAGCAGGAGGATCTTGGG	This study
MDR1-EQEQ-mutF2	GCTGGATCAGGCCACGTCAGCCTTGGACAC	This study
MDR1-EQEQ-mutR2	CGTGGCTTGATCCAAAAGCAAAATATG	This study

Supplementary References

1. Kodan, A. *et al.* Structural basis for gating mechanisms of a eukaryotic P-glycoprotein homolog. *Proc. Natl. Acad. Sci. U S A* **111**, 4049–4054 (2014).
2. Sato, T. *et al.* Functional role of the linker region in purified human P-glycoprotein. *FEBS J.* **276**, 3504–3516 (2009).
3. Lerner-Marmarosh, N., Gimi, K., Urbatsch, I. L., Gros, P. & Senior, A. E. Large scale purification of detergent-soluble P-glycoprotein from Pichia pastoris cells and characterization of nucleotide binding properties of wild-type, Walker A, and Walker B mutant proteins. *J. Biol. Chem.* **274**, 34711–34718 (1999).
4. Kim, Y. & Chen, J. Molecular structure of human P-glycoprotein in the ATP-bound, outward-facing conformation. *Science* **359**, 915–919 (2018).
5. Dawson, R. J. & Locher, K. P. Structure of a bacterial multidrug ABC transporter. *Nature* **443**, 180–185 (2006).
6. Ward, A., Reyes, C. L., Yu, J., Roth, C. B. & Chang, G. Flexibility in the ABC transporter MsbA: Alternating access with a twist. *Proc. Natl. Acad. Sci. U S A* **104**, 19005–19010 (2007).
7. Aller, S. G. *et al.* Structure of P-glycoprotein reveals a molecular basis for poly-specific drug binding. *Science* **323**, 1718–1722 (2009).
8. Jin, M. S., Oldham, M. L., Zhang, Q. & Chen, J. Crystal structure of the multidrug transporter P-glycoprotein from Caenorhabditis elegans. *Nature* **490**, 566–569 (2012).
9. Russ, W. P. & Engelman, D. M. The GxxxG motif: a framework for transmembrane helix-helix association. *J. Mol. Biol.* **296**, 911–919 (2000).
10. Procko, E., Ferrin-O'Connell, I., Ng, S. L. & Gaudet, R. Distinct structural and functional properties of the ATPase sites in an asymmetric ABC transporter. *Mol. Cell* **24**, 51–62 (2006).
11. Nakamura, K. *et al.* Functional expression of Candida albicans drug efflux pump Cdr1p in a Saccharomyces cerevisiae strain deficient in membrane transporters. *Antimicrob. Agents Chemother.* **45**, 3366–3374 (2001).
