# Supplementary material for: Inward- and outward-facing X-ray crystal structures of homodimeric P-glycoprotein CmABCB1
Source: Nat Commun. 2019 Jan 8;10:88. doi: 10.1038/s41467-018-08007-x (PMC6325147; doi:10.1038/s41467-018-08007-x)
Supplement: Supplementary file 4 — Description of Additional Supplementary Files [file 41467_2018_8007_MOESM4_ESM.docx]

**Title:** Supplementary Movie 1.
**Description:** Animation illustrating the overall structural change. The structures of QTA CmABCB1 in the inwardand outward-facing states were aligned based on their overall homodimer structures, and morphing movies were generated using the ‘morph’ command of PyMOL 1.8. The movie shows that the movements involved in the structural change consist of four elements: rotation, translation, tilt, and twist.

**Title:** Supplementary Movie 2.
**Description:**  Animation illustrating the structural change of TMD propagated upon Mg•AMP-PNP binding at NBD dimer. The structures of QTA CmABCB1 in the inward- and outward-facing states were aligned based on their overall homodimer structures, and morphing movies were generated using the ‘morph’ command of PyMOL 1.8.
