# Supplementary material for: Inward- and outward-facing X-ray crystal structures of homodimeric P-glycoprotein CmABCB1
Source: Nat Commun. 2019 Jan 8;10:88. doi: 10.1038/s41467-018-08007-x (PMC6325147; doi:10.1038/s41467-018-08007-x)
Supplement: Supplementary file 6 — Reporting Summary [file 41467_2018_8007_MOESM6_ESM.pdf]

natureresearch

Corresponding author(s): Hiroaki Kato

Reporting Summary

Nature Research wishes to improve the reproducibility of the work that we publish. This form provides structure for consistency and transparency in reporting. For further information on Nature Research policies, see Authors & Referees and the Editorial Policy Checklist.

Statistical parameters

When statistical analyses are reported, confirm that the following items are present in the relevant location (e.g. figure legend, table legend, main text, or Methods section).

n/a	Confirmed	
☐	☒	The exact sample size (*n*) for each experimental group/condition, given as a discrete number and unit of measurement
☐	☒	An indication of whether measurements were taken from distinct samples or whether the same sample was measured repeatedly
☒	☐	The statistical test(s) used AND whether they are one- or two-sided<br>*Only common tests should be described solely by name; describe more complex techniques in the Methods section.*
☐	☒	A description of all covariates tested
☒	☐	A description of any assumptions or corrections, such as tests of normality and adjustment for multiple comparisons
☐	☒	A full description of the statistics including central tendency (e.g. means) or other basic estimates (e.g. regression coefficient) AND variation (e.g. standard deviation) or associated estimates of uncertainty (e.g. confidence intervals)
☒	☐	For null hypothesis testing, the test statistic (e.g. *F*, *t*, *r*) with confidence intervals, effect sizes, degrees of freedom and *P* value noted<br>*Give P values as exact values whenever suitable.*
☒	☐	For Bayesian analysis, information on the choice of priors and Markov chain Monte Carlo settings
☒	☐	For hierarchical and complex designs, identification of the appropriate level for tests and full reporting of outcomes
☒	☐	Estimates of effect sizes (e.g. Cohen's *d*, Pearson's *r*), indicating how they were calculated
☐	☒	Clearly defined error bars<br>*State explicitly what error bars represent (e.g. SD, SE, CI)*

Our web collection on statistics for biologists may be useful.

Software and code

Policy information about availability of computer code

Data collection	XDS, HKL2000
Data analysis	COOT, REFMAC5, PHENIX, PyMOL, GRAFIT, KaleidaGraph

For manuscripts utilizing custom algorithms or software that are central to the research but not yet described in published literature, software must be made available to editors/reviewers upon request. We strongly encourage code deposition in a community repository (e.g. GitHub). See the Nature Research guidelines for submitting code & software for further information.

Data

Policy information about availability of data

All manuscripts must include a data availability statement. This statement should provide the following information, where applicable:

- Accession codes, unique identifiers, or web links for publicly available datasets
- A list of figures that have associated raw data
- A description of any restrictions on data availability

6A6M, 6A6N

Field-specific reporting

Please select the best fit for your research. If you are not sure, read the appropriate sections before making your selection.

- [x] Life sciences
- [ ] Behavioural & social sciences
- [ ] Ecological, evolutionary & environmental sciences

For a reference copy of the document with all sections, see nature.com/authors/policies/ReportingSummary-flat.pdf

Life sciences study design

All studies must disclose on these points even when the disclosure is negative.

Sample size	The sample size was determined in consideration of calculating standard error.
Data exclusions	No data were excluded.
Replication	All attempts at replication were successful.
Randomization	All data were used.
Blinding	Blinding was not relevant because this study did not involve animals and human research participants.

Reporting for specific materials, systems and methods

Materials & experimental systems

n/a	Involved in the study
[x]	[ ] Unique biological materials
[ ]	[x] Antibodies
[ ]	[x] Eukaryotic cell lines
[x]	[ ] Palaeontology
[x]	[ ] Animals and other organisms
[x]	[ ] Human research participants

Methods

n/a	Involved in the study
[x]	[ ] ChIP-seq
[x]	[ ] Flow cytometry
[x]	[ ] MRI-based neuroimaging

Antibodies

Antibodies used	Penta His HRP conjugate(Qiagen, Mat#1014992), Mouse monoclonal anti-GAPDH(ThermoFisher, Cat#MA5-15738), Goat anti-mouse IgG-AP conjugate(BioRad, Cat#170-6520)
Validation	The antibodies were validated by lack of immunostaining of cell extracts that do not express the recognized antigen (that is, mock transfected cells).

Eukaryotic cell lines

Policy information about cell lines

Cell line source(s)	FreeStyle 293-F cell (ThermoFisher, previously Invitrogen, R79007)
Authentication	FreeStyle 293-F cell was purchased from Invitrogen and used without authentication.
Mycoplasma contamination	The cell line was not tested for mycoplasma contamination.
Commonly misidentified lines (See ICLAC register)	FreeStyle 293-F cells were used only for recombinant protein production and purification (supplementary figure 5D and 4E). FreeStyle 293-F cell is not listed commonly misidentified cell lines.
